# *α*-MSH Influences the Excitability of Feeding-Related Neurons in the Hypothalamus and Dorsal Vagal Complex of Rats

**DOI:** 10.1155/2017/2034691

**Published:** 2017-11-26

**Authors:** Hong-Zai Guan, Jing Dong, Zheng-Yao Jiang, Xi Chen

**Affiliations:** ^1^Department of Clinical Hematology, Medical College, Qingdao University, Qingdao, China; ^2^Department of Special Medicine, Medical College, Qingdao University, Qingdao, China; ^3^Department of Physiology, Shandong Provincial Key Laboratory of Pathogenesis and Prevention of Neurological Disorders and State Key Disciplines: Physiology, Medical College, Qingdao University, Qingdao, China

## Abstract

Alpha-melanocyte-stimulating hormone (*α*-MSH) is processed from proopiomelanocortin (POMC) and acts on the melanocortin receptors, MC3 and MC4. *α*-MSH plays a key role in energy homeostasis. In the present study, to shed light on the mechanisms by which *α*-MSH exerts its anorectic effects, extracellular neuronal activity was recorded in the hypothalamus and the dorsal vagal complex (DVC) of anesthetized rats. We examined the impact of *α*-MSH on glucose-sensing neurons and gastric distension (GD) sensitive neurons. In the lateral hypothalamus (LHA), *α*-MSH inhibited 75.0% of the glucose-inhibited (GI) neurons. In the ventromedial nucleus (VMN), most glucose-sensitive neurons were glucose-excited (GE) neurons, which were mainly activated by *α*-MSH. In the paraventricular nucleus (PVN), *α*-MSH suppressed the majority of GI neurons and excited most GE neurons. In the DVC, among the 20 GI neurons examined for a response to *α*-MSH, 1 was activated, 16 were depressed, and 3 failed to respond. Nineteen of 24 GE neurons were activated by *α*-MSH administration. Additionally, among the 42 DVC neurons examined for responses to GD, 23 were excited (GD-EXC) and 19 were inhibited (GD-INH). Fifteen of 20 GD-EXC neurons were excited, whereas 11 out of 14 GD-INH neurons were suppressed by *α*-MSH. All these responses were abolished by pretreatment with the MC3/4R antagonist, SHU9119. In conclusion, the activity of glucose-sensitive neurons and GD-sensitive neurons in the hypothalamus and DVC can be modulated by *α*-MSH.

## 1. Introduction

Alpha-melanocyte-stimulating hormone (*α*-MSH) is a 13-amino-acid peptide derived from proopiomelanocortin (POMC) [1]. It is mainly expressed in the nucleus of the solitary tract (NTS), the pituitary gland, the arcuate nucleus (ARC), and other peripheral tissues [2]. It has been shown that *α*-MSH decreases food intake or body weight after infusion into the lateral [3] and fourth brain ventricles (4v) [4], the paraventricular nucleus (PVN), and the ARC [5]. *α*-MSH regulates food intake and energy homeostasis by acting on melanocortin-3 receptors (MC3R) and melanocortin-4 receptors (MC4R). MC3Rs are distributed throughout the hypothalamus and limbic structures, while MC4Rs are more extensively expressed in the amygdala, thalamus, cortex, striatum, hippocampus, hypothalamus, notably, the ARC, the PVN, the LHA (lateral hypothalamic area), the VMN (ventromedial hypothalamic nucleus), and the brainstem (notably in the dorsomotor nucleus of the vagus (DMNV), the NTS, and the parabrachial nucleus) [6, 7]. Blockade of melanocortin receptors (e.g., by MC3/4R antagonist SHU-9119) increases food intake and body weight [8].

In the CNS, glucose-sensing neurons monitor blood glucose levels and initiate glucoprivic feeding. There are two types of glucose-sensing neurons: glucose-excited (GE) neurons and glucose-inhibited (GI) neurons, which increase and decrease their firing rates, respectively, in response to rising extracellular glucose concentrations [9, 10] in the hypothalamus and the dorsal vagal complex (DVC). The DVC is composed of 3 parts: the area postrema (AP), the NTS, and the DMNV in the brain stem. The NTS also contains another type of feeding-related neurons, gastric distension (GD) sensitive neurons, which alter their firing rate in response to GD. In fact, the caudal brainstem receives direct neural and humoral feedback signals from the gut [11–13]. GD, a type of satiation signal that postprandially imitates the physiological effect existing in the gastrointestinal system, reaches the NTS via vagal afferent fibers.

In the present study, to further clarify the mechanism underlying the anorectic effects of *α*-MSH, we identified glucose-sensing neurons and GD-sensitive neurons in the hypothalamus and the brainstem and examined the changes in their firing rates in response to local administration of *α*-MSH.

## 2. Materials and Methods

### 2.1. Animals

Adult Wistar rats (Qingdao Institute for Drug Control) weighing 220–280 g of either sex were used. Housing conditions, such as humidity, temperature (22 ± 2°C), and illumination (12 : 12 h light/dark cycle, lights on/off: 8:00 a.m./8:00 p.m.) were controlled for at least 7 days prior to the experiments. Standard laboratory chow pellets and tap water were available ad libitum. All animal experiments followed the ethical guidelines of Qingdao University for animal care. During the experiments, rectal temperature was maintained between 36 and 38°C using a thermostatically controlled heating pad.

### 2.2. Extracellular Electrophysiological Recordings

The tested rats were anesthetized with urethane (1.0 g/kg, i.p.), and a maintenance dose of anesthetic was administered when necessary. A stereotaxic apparatus (Narishige SN-3, Tokyo, Japan) with an incisor bar 3.3 mm below the center of the ear bars was used for immobilizing anesthetized animals. The stereotaxic coordinates [14] were as follows: LHA (1.8–3.2 mm caudal to the bregma, 1.5–2.5 mm lateral to the sagittal sinus, and 7.5–9.0 mm below the surface of the skull); VMN (1.8–3.1 mm caudal to the bregma, 0.2–1.0 mm lateral to the sagittal sinus, and 9.3–10.0 mm below the surface of the skull); PVN (1.6–1.9 mm caudal and 0.1–0.7 mm lateral to the bregma, 7.7–8.4 mm below the surface of the skull); DVC (0.2–1.0 mm lateral from midline, rostral 1.0 mm, 0.5 mm caudal from the obex, and 0.5–1.8 mm deep from the medulla dorsal surface). Portions of the skull and cerebellum were removed to expose the brain. The dura mater and arachnoid of the exposed area were carefully removed and then covered with warm agar (3-4% in saline) to maintain neuronal recording stability.

Gastric distension (GD) was induced by a latex balloon attached to polyethylene tubing (PE-240) surgically placed in the stomach [15]. Gastric contents were promptly washed out with warm isotonic saline through a small incision in the fundus wall after midline laparotomy. Gastric distension was kept at a constant volume for 10–30 s by increasing the balloon to a 1.5–2.0 ml volume at an inflation rate of 0.5 ml/s. There was no effect on arterial blood pressure at these levels. To prevent duodenal reflux from affecting the gastric volume, the pylorus was ligatured. Electrophysiological recordings and micropressure injections were recorded using four-barrel glass microelectrodes with a 3–10 *μ*m tip diameter and 5–20 MΩ resistance. Three of these barrels were connected to a 4-channel pressure injector (PM2000B, Micro Data Instrument, Inc., USA) filled with 5 Mm glucose solution (PH 7.4), 500 nM *α*-MSH solution (Sigma, M4135), and 1 mM SHU9119 solution (Sigma, M4603), which were separately dissolved in 0.9% NaCl. The other glass microelectrode was used for recording and was filled with 0.5 M sodium acetate and 2% pontamine sky blue. Drugs were ejected onto the surface of firing cells using a short pulse gas pressure (1500 ms, 5.0–15.0 psi). The intrabarrel drug concentrations were varied based on their efficacy of altering cell firing. Less than 1 nl *α*-MSH was applied to the firing cells during extracellular recording.

The recorded electrical signals, amplified and displayed on a memory oscilloscope (VC-11, Nihon Kohden), were fed into a signal analyzer. The computer incorporated a signal discriminator and allowed unitary data to be stored online.

### 2.3. Statistical Analysis

The data were expressed as the mean ± standard error of the mean (SEM). Using Student's *t*-test, the mean discharge frequencies were compared with the last 120 s immediately prior to treatment when computer graphs indicated that the modification of neuronal activity lasted more than 50 seconds during and/or after reagent ejection. Changes in neuronal firing frequencies were considered to be responses if they were statistically significant (*P* < 0.05) and exceeded 20%.

## 3. Results

### 3.1. *α*-MSH Suppresses Neuronal Activity of LHA GI Neurons

**Figure 1:**
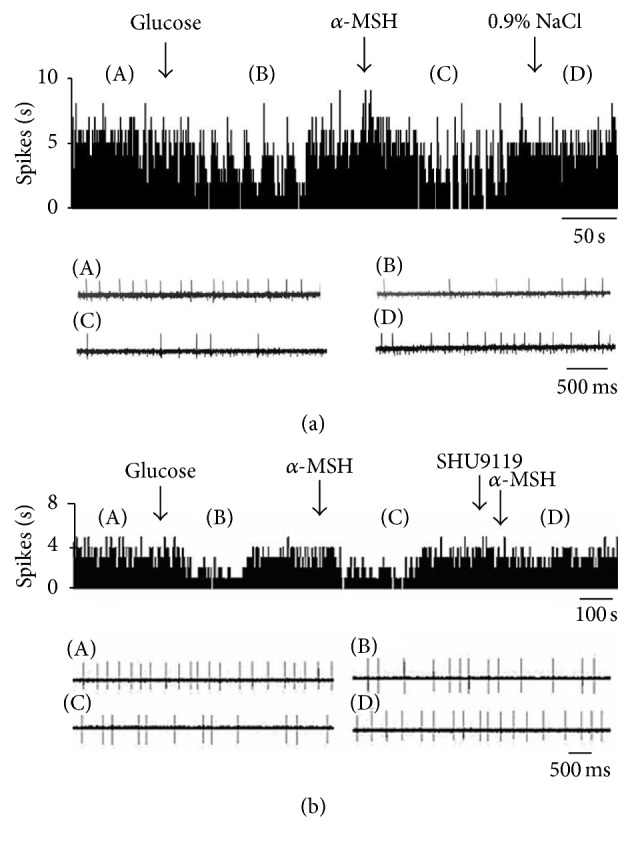
Effect of *α*-MSH on GI neurons in the LHA. (a) Typical frequency histograms show that GI neurons were inhibited by *α*-MSH. The first arrow indicates 5 mM glucose, the second arrow indicates *α*-MSH (500 nM), and the third arrow indicates a 0.9% NaCl treated control. (b) Typical frequency histograms show that SHU9119 partially abolished the effects of *α*-MSH on GI neurons in the LHA. The first arrow indicates 5 mM glucose, the second arrow indicates *α*-MSH (500 nM), the third arrow indicates pretreatment with SHU9119 (1 *μ*M), and the fourth arrow indicates another injection of *α*-MSH.

Spontaneously firing units were recorded before, during, and after glucose/*α*-MSH was applied by microinjection via four-barrel glass microelectrodes connected to a 4-channel pressure injector. Among the 62 total neurons in the LHA, 26 were identified as GE neurons that increased their firing rate after an injection of glucose. Of the 26 GE neurons, 8 were excited, 8 were inhibited, and 10 showed no response to *α*-MSH (Table 1). Thirty-six discharging neurons were inhibited by glucose and identified as GI neurons. The main effect after intra-LHA administration of *α*-MSH to GI neurons (27/36, 75.0%) was inhibition (Figure 1). Administration of nesfatin-1 significantly decreased the firing rate of GI neurons from 3.72 ± 0.49 Hz to 1.39 ± 0.29 Hz (*n* = 27, *P* < 0.01) (Figure 5(a)). In 19 GI neurons, the change in the firing rate decreased from 56.36 ± 6.22% to 11.51 ± 2.18% after pretreatment with SHU9119. This indicated that the effect of *α*-MSH was partially abolished by SHU9119 (Figure 1). *α*-MSH exhibited different effects on GE and GI neurons (*P* < 0.01).

### 3.2. The Effect of *α*-MSH on Glucose GE and GI Neurons in the VMN

**Figure 2:**
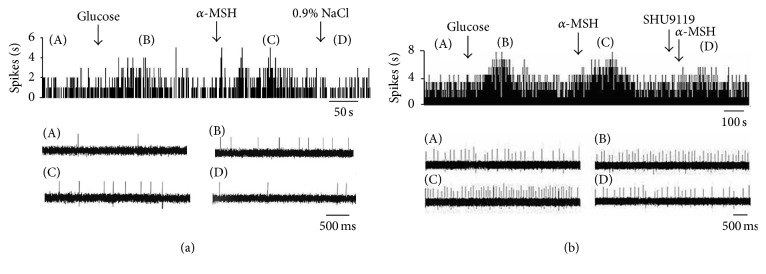
Effect of *α*-MSH on GE neurons in the VMN. (a) Typical frequency histograms show that GE neurons were activated by *α*-MSH. (b) Typical frequency histograms show that SHU9119 partially abolished the effects of *α*-MSH on GE neurons in the VMN.

Of 30 glucose-sensitive neurons in the VMN, 73.3% (22/30) were excited (GE) and 26.7% (8/30) were inhibited (GI). Of those examined for a response to *α*-MSH, 1 was activated and the others were depressed (Table 1). Of the 22 GE neurons, 20 were excited by *α*-MSH (Figure 2), increasing their firing rate from 1.59 ± 0.19 Hz to 3.93 ± 0.32 Hz (*P* < 0.01) (Figure 5(b)). Pretreatment with SHU9119 blocked the enhancement of *α*-MSH (Figure 2).

### 3.3. The Effect of *α*-MSH on Glucose GE and GI Neurons in the PVN

**Figure 3:**
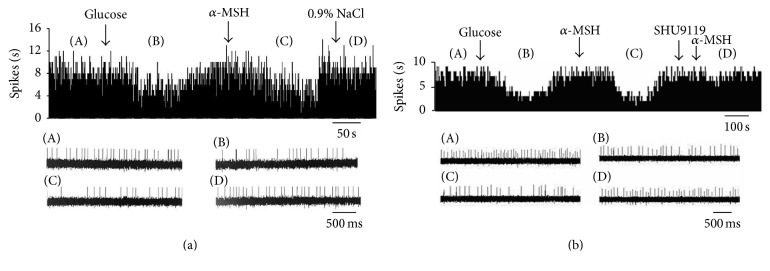
Effect of *α*-MSH on GI neurons in the PVN. (a) Typical frequency histograms show that GI neurons were inhibited by *α*-MSH. (b) Typical frequency histograms show that SHU9119 partially abolished the effects of *α*-MSH on GI neurons in the PVN.

**Figure 4:**
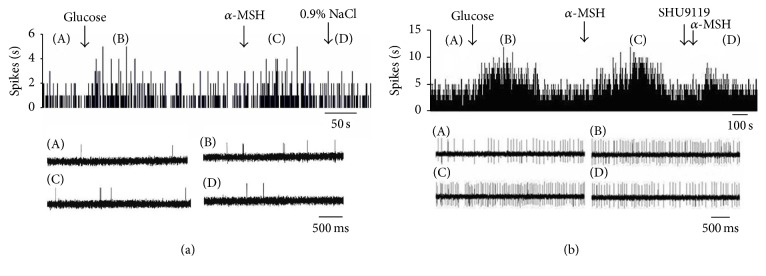
Effect of *α*-MSH on GE neurons in the PVN. (a) Typical frequency histograms show that GE neurons were activated by *α*-MSH. (b) Typical frequency histograms show that SHU9119 partially abolished the effects of *α*-MSH on GE neurons in the PVN.

**Figure 5:**
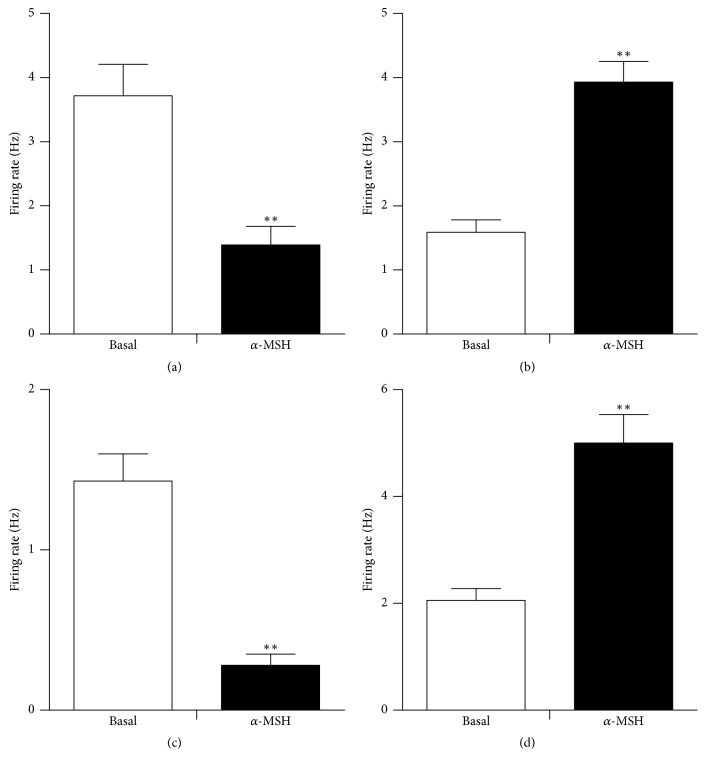
The discharge frequency change in hypothalamic glucose-sensitive neurons after injection of *α*-MSH. (a) GI neurons in the LHA; (b) GE neurons in the VMN; (c) GI and (d) GE neurons in the PVN. ^*∗∗*^*P* < 0.01.

Among the 47 recorded neurons that responded to glucose, 27 were activated by glucose, identified as GE neurons, and the other 20 were suppressed, identified as GI neurons. Of the 20 GI neurons examined for responses to *α*-MSH, 6 were excited and 14 were inhibited (Table 1, Figures 3 and 5(c)). After injection of glucose, the firing rate of the GE neurons increased from 3.34 ± 0.65 Hz to 6.12 ± 0.94 Hz (*P* < 0.01). In the 27 GE neurons, 88.9% (24/27) were excited, and 11.1% (3/27) were inhibited by injection of *α*-MSH. The main effect of intra-PVN administration of *α*-MSH into GE neurons was excitatory. Twenty-four of the 27 GE neurons increased their firing rate by 156.5 ± 19.7% (from 2.06 ± 0.22 Hz to 5.00 ± 0.54 Hz, *P* < 0.01) after *α*-MSH was locally injected (Figure 5(d)). Injection of the MC4R antagonist, SHU9119, weakened the effect of *α*-MSH (Figure 4). Therefore, *α*-MSH excited most GE neurons and inhibited most GI neurons (*P* < 0.01).

### 3.4. The Effects of Glucose and *α*-MSH Applied Locally in the DVC

**Figure 6:**
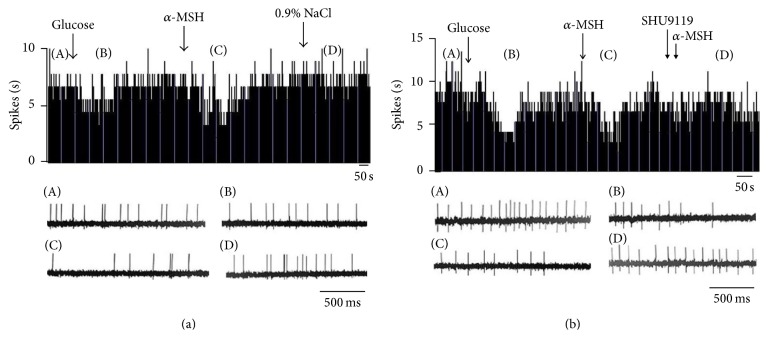
Effect of *α*-MSH on GI neurons in the DVC. (a) Typical frequency histograms show that GI neurons were inhibited by *α*-MSH. (b) Typical frequency histograms show that SHU9119 partially abolished the effects of *α*-MSH on GI neurons in the DVC.

**Figure 7:**
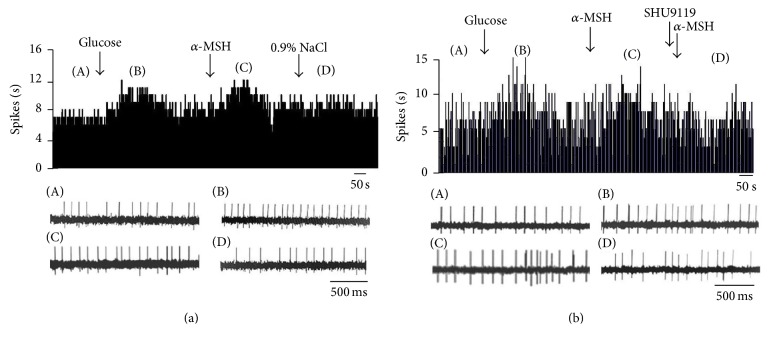
Effect of *α*-MSH on GE neurons in the DVC. (a) Typical frequency histograms show that GE neurons were activated by *α*-MSH. (b) Typical frequency histograms show that SHU9119 partially abolished the effects of *α*-MSH on GE neurons in the DVC.

Of the total neurons, 107 were tested with glucose in the DVC. Excitation and inhibition were observed in response to glucose injected by micropressure. Twenty were depressed by glucose and identified as glucose-INH neurons; 24 were activated by glucose and identified as glucose-EXC neurons, and 51 failed to respond to glucose (Table 2). After glucose injection, the firing rate of the GI neurons decreased from 8.23 ± 1.32 Hz to 4.45 ± 0.83 Hz (*P* < 0.01). Of the 20 GI neurons in the DVC examined for a response to *α*-MSH, 16 were depressed, 1 was activated, and 3 failed to respond. For the 16 GI neurons suppressed by *α*-MSH, the firing rate decreased from 6.78 ± 1.04 Hz to 3.47 ± 0.67 Hz (*P* < 0.01) (Figures 6 and 10(a)). For the 19 out of 24 GE neurons activated by *α*-MSH, the firing rate increased by 51.77 ± 10.30% (from 9.76 ± 1.95 Hz to 14.84 ± 3.41 Hz, *P* < 0.05) (Figures 7 and 10(b)). *α*-MSH had no effect on the remaining 5 GE neurons. The responses induced by *α*-MSH were abolished by SHU9119 pretreatment. A control injection with 0.9% NaCl had no effect on these neurons, in contrast to the responses to *α*-MSH.

### 3.5. The Effect of *α*-MSH on GD-Sensitive Neurons in the DVC

**Figure 8:**
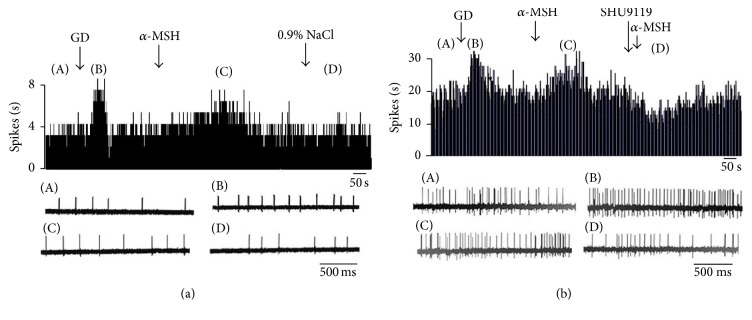
Effect of *α*-MSH on GD-EXC neurons in the DVC. (a) Typical frequency histograms show that GD-EXC neurons were activated by *α*-MSH. The first arrow indicates gastric distention, the second arrow indicates *α*-MSH (500 nM), and the third arrow indicates a 0.9% NaCl treated control. (b) Typical frequency histograms show that SHU9119 partially abolished the effects of *α*-MSH on GD-EXC neurons in the DVC. The first arrow indicates gastric distention, the second arrow indicates *α*-MSH (500 nM), the third arrow indicates pretreatment with SHU9119 (1 *μ*M), and the fourth arrow indicates another injection of *α*-MSH.

**Figure 9:**
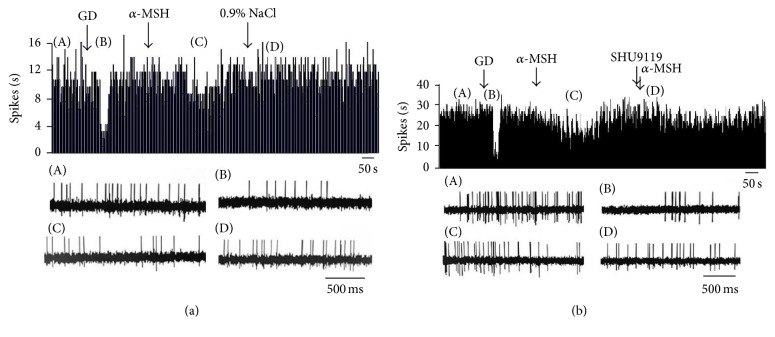
Effect of *α*-MSH on GD-INH neurons in the DVC. (a) Typical frequency histograms show that GD-INH neurons were suppressed by *α*-MSH. (b) Typical frequency histograms show that SHU9119 partially abolished the effects of *α*-MSH on GD-INH neurons in the DVC.

**Figure 10:**
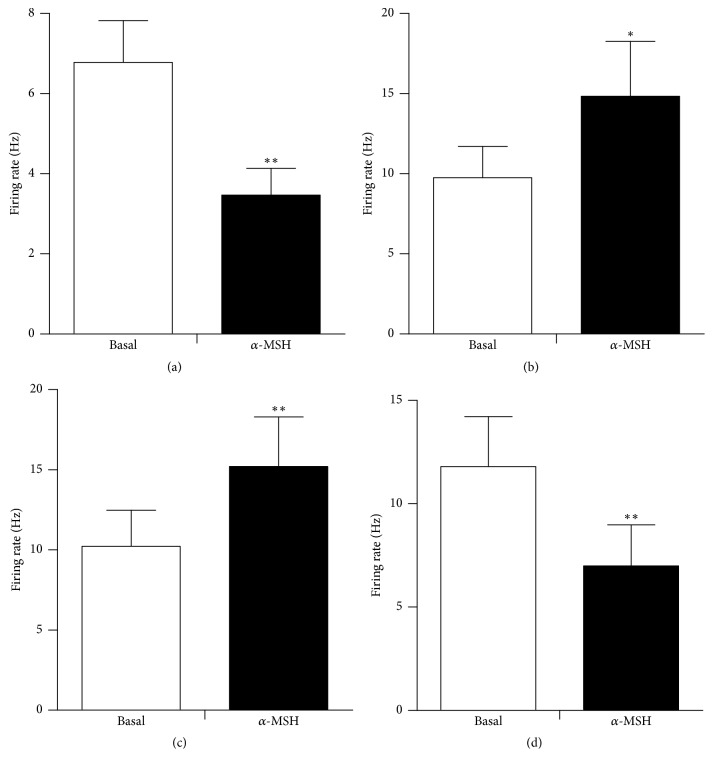
The change in the firing rate of DVC neurons after injection of *α*-MSH. (a) GI neurons, (b) GE neurons, (c) GD-EXC neurons, and (d) GD-INH neurons. ^*∗*^*P* < 0.05; ^*∗∗*^*P* < 0.01.

Among the 42 DVC neurons examined for responses to gastric distension (GD), 20 were excited (GD-EXC), and 14 were inhibited (GD-INH) (Table 2). *α*-MSH exhibited different effects on GD-EXC and GD-INH neurons (*P* < 0.01). The firing rate of the GD-EXC neurons increased from 9.52 ± 1.96 Hz to 14.73 ± 2.38 Hz (*P* < 0.01), whereas the firing rate of the GD-INH neurons decreased from 9.22 ± 1.63 Hz to 5.89 ± 1.18 Hz (*P* < 0.01). Among the 20 GD-EXC neurons examined, 15 were activated (from 10.23 ± 2.24 Hz to 15.21 ± 3.09 Hz, *P* < 0.01) (Figure 10(c)), and 5 exhibited no change after the administration of *α*-MSH (Figure 8). Of the 14 GD-INH neurons tested with *α*-MSH, 11 were suppressed (from 11.81 ± 2.41 Hz to 6.99 ± 1.99 Hz, *P* < 0.01) (Figures 9 and 10(d)), and 3 exhibited no effect. After pretreatment with SHU9119, injection of *α*-MSH failed to cause any changes in the firing rate.

## 4. Discussion

In the present study, our results showed that *α*-MSH exerted similar effects on glucose-sensitive neurons in the hypothalamus and the DVC: GE neurons were excited, while GI neurons were inhibited by *α*-MSH. These responses were blocked by pretreatment with the melanocortin receptor antagonist SHU-9119. In addition, *α*-MSH modulated the activity of GD-sensitive neurons in the DVC; GD-EXC neurons increased and GD-INH neurons decreased their firing rates after injection of *α*-MSH.

Our data showed that LHA and VMN contain more GI and GE neurons, respectively, while the PVN contains both GI and GE neurons. In agreement with our results, Silver and Erecinska [16] demonstrated that increased plasma glucose inhibited the majority of LHA glucose-sensing neurons (GI neurons) and in the VMH 43% of neurons increased their action potential frequency as glucose levels increased, which were identified as GE neurons. Melnick et al. found that, in the PVN, approximately 24% of neurons were GE neurons; approximately 26% of neurons were GI neurons in brain slices [17]. The MC3Rs/MC4Rs distributed in hypothalamic nuclei are implicated in feeding control. Previous studies have shown that intracerebroventricularly administered MC4R antagonists acutely stimulate food intake, and chronic delivery of *α*-MSH in the rat hypothalamus reduces food intake and body weight [18]. Within the LHA, neurons expressing the wake-promoting peptides, orexins, are GI neurons, whereas neurons expressing the sleep-promoting neuropeptide melanin-concentrating hormone (MCH) are GE neurons [19, 20]. In addition to orexin neurons, GI neurons also overlap with the NPY neurons of the LHA [21]. Both orexin and NPY serve as potent orexigenic signals in the hypothalamus. Our results showed that *α*-MSH inhibited most GI neurons in the LHA, which may be responsible for the inhibition of food intake. Li et al. showed that *α*-MSH exerted both inhibitory and excitatory effects on VMN neurons in brain slices [22], while Fu and Van Den Pol showed that MC3/4 receptor agonists only inhibited glutamatergic neurons in the VMN [23]. In the present study, we found that *α*-MSH exerted bidirectional effects on glucose-sensitive neurons. Brain-derived neurotrophic factor (BDNF), which decreases food intake after central and peripheral administration, is abundantly expressed in the VMN. It serves as a downstream target of MC4R-mediated signaling and participates in the regulation of energy balance and feeding behavior [24]. It has been shown that *α*-MSH activates PVN-firing MC4R neurons in brain slices by closing the inwardly rectifying potassium channel, Kir7.1, via the G*α*s-coupled signaling pathway [25]. We observed that *α*-MSH administration excited most spontaneously discharging GE neurons in the PVN, indicating glucose-activated neurons that express MC4R. In addition, PVN is abundant in an anorexigenic peptide, nesfatin-1. Nesfatinergic neurons can be activated by fibroblast growth factor 21 with elevated glucose, which indicates the close correlation between glucose and peptides [26]. Moreover, *α*-MSH inactivated the majority of GI neurons in vivo, indicating either an indirect effect via MC4R or an effect due to the different electrical recording methods. It would be helpful to explain the different effects of *α*-MSH on GE and GI neurons if the neuropeptides or transmitters containing in glucose-sensitive neurons are completely clear.

In the DVC, we found that most GE neurons (19/24) were excited and most GI neurons (16/20) were inhibited by administration of *α*-MSH. Mimee and Ferguson noted that 75% of GE neurons were depolarized by *α*-MSH [27], which was consistent with our results. However, 29% of the GI neurons were hyperpolarized, which differs from our results. Campos et al. noted that activation of NTS MC4Rs reduced food intake by PKA-catalyzed phosphorylation of synapsin I and, thereby, increased glutamatergic neurotransmission from vagal afferent endings [28]. As Mimee and Ferguson noted, NTS GE neurons are GABAergic, and activation of NTS GE neurons leads to decreased gastric motility, while activation of a subset of GI NTS neurons results in glucagon secretion. MC4Rs are detected in the autonomic circuitry involved in gastric function, especially in the dorsal motor nucleus of the vagus nerve (DMV) [29], which indicates the participation of *α*-MSH in the modulation of gastric activity by the melanocortinergic-sympathetic pathway or melanocortinergic-parasympathetic pathway. GD mimics the postprandial gastric signal and activates mechanosensitive tension receptors, which relay information through the vagal and splanchnic nerves to the DVC. Our results showed that GD-sensitive neurons responded to *α*-MSH, indicating that GD may inhibit food intake through activation of MC4R neurons in the DVC. *α*-MSH influences the excitability of glucose, and GD-sensitive neurons integrate a variety of hormonal, metabolic, transmitter peptide, or mechanical signals that function to reduce food intake and body weight.

## 5. Conclusion

Our current findings suggest that the anorectic effects of *α*-MSH are partly modulated by the excitability of glucose-sensing neurons in the forebrain and hindbrain.

## Figures and Tables

**Table 1:** Effects of *α*-MSH on glucose-sensitive neurons in the hypothalamus.

	Response to glucose	Response to *α*-MSH		
		Activated	Depressed	Insensitive
LHA	26 GE neurons	8 (30.8%)	8 (30.8%)	10 (38.4%)
	36 GI neurons	4 (11.1%)	27 (75.0%)	5 (13.9%)
VMN	22 GE neurons	20 (90.0%)	1 (4.5%)	1 (4.5%)
	8 GI neurons	1 (12.5%)	7 (87.5%)	0 (0.0%)
PVN	27 GE neurons	24 (88.9%)	3 (11.1%)	0 (0.0%)
	20 GI neurons	6 (30.0%)	14 (70.0%)	0 (0.0%)

**Table 2:** Effects of *α*-MSH on glucose-sensitive and GD-sensitive neurons in the DVC.

		Response to *α*-MSH		
		Activated	Depressed	Insensitive
Response to glucose	24 GE neurons	19 (79.2%)	0 (0.0%)	5 (20.8%)
	20 GI neurons	1 (5.0%)	16 (80.0%)	3 (15.0%)
Response to GD	20 GD-EXC	15 (75.0%)	0 (0.0%)	5 (25.0%)
	14 GD-INH	0 (0.0%)	11 (78.6%)	3 (21.4%)
